# Motifs in the amino-terminus of CENP-A are required for its accumulation within the nucleus and at the centromere

**DOI:** 10.18632/oncotarget.17204

**Published:** 2017-04-18

**Authors:** Ruiqi Jing, Jiajie Xi, Ye Leng, Wen Chen, Guiying Wang, Wenwen Jia, Jiuhong Kang, Songcheng Zhu

**Affiliations:** ^1^ Clinical and Translational Research Center of Shanghai First Maternity and Infant Health Hospital, Shanghai Key Laboratory of Signaling and Disease Research, Collaborative Innovation Center for Brain Science, School of Life Science and Technology, Tongji University, Shanghai, 200092, China

**Keywords:** centromeric protein A (CENP-A), histone variant, nuclear localization, centromeric localization

## Abstract

Centromere protein A (CENP-A) is a variant of core histone H3 that marks the centromere's location on the chromosome. The mechanisms that target the protein to the nucleus and the centromere have not been defined. In this study, we found that deletion of the first 53 but not the first 29 residues of CENP-A from the amino-terminus, resulted in its cytoplasmic localization. Two motifs, R^42^R^43^R^44^ and K^49^R^52^K^53^K^56^, which are reported to be required for DNA contact in the centromere nucleosome, were found to be critical for CENP-A nuclear accumulation. These two motifs potentially mediated its interaction with Importin-β but were not involved in CENP-A centromeric localization. A third novel motif, L^60^L^61^I^62^R^63^K^64^, was found to be essential for the centromeric accumulation of CENP-A. The nonpolar hydrophobic residues L^60^L^61^I^62^, but not the basic residues R^63^K^64^, were found to be the most important residues. A protein interaction assay suggested that this motif is not involved in the interaction of CENP-A with its deposition factors but potentially mediates its interaction with core histone H4 and CENP-B. Our study uncovered the role of the amino-terminus of CENP-A in localization.

## INTRODUCTION

Chromatin is organized in arrays of nucleosomes in which the core histones, H2A, H2B, H3 and H4, are arranged as an octameric core around which DNA is wrapped. The linker histones H1 bind to the linker DNA connecting adjacent nucleosomes [1]. In addition to the above major histone types, many histone variants have been found, including H2A variants H2A.Z, MacroH2A, H2A-Bbd, H2AvD, and H2A.X; H3 variants H3.3 and centromeric H3 (CenH3) [1]. The similarity between the major histone subtypes and the variants range from almost no amino acid differences to extremely divergent changes [2]. Histones are functionally conserved as indicated by their high degree of structural conservation [3]. Each histone contains a conserved C-terminal histone fold domain (HFD) and a less structured and unique amino-terminus, commonly referred to as an ‘N-tail’. Histones are highly basic proteins and the ‘N-tail’ provides the majority of the basic amino acids Arg (R) and Lys (K) to the protein. The amino-terminus of histones is subjected to a wide variety of post-translational modifications, most of which occur on the Arg and Lys residues. The combinations of modifications on Arg or Lys and the Ser/Thr residues at the amino-terminus are thought to constitute a code directing distinct structural states of chromatin [4, 5].

The minor histone variant forms can replace the corresponding major histone in the nucleosome and carry out specific functions [6, 7]. The incorporation of different species of histone variants into nucleosomes provides further differentiation and epigenetic chromatin diversity [1, 8]. The differentiation and specific functions of chromatin directed by a histone variant is especially conspicuous at centromeres, where the H3 variant, CENP-A, is assembled into specialized nucleosomes that form the foundation for the kinetochore assembly [9–11]. The crystal structure of CENP-A revealed that it is quite similar to the structure of histone H3 variants and consists of an unstructured amino terminal αN-helix, α1 helix, Loop1, β1-sheet, β2-sheet, α2 helix, and α3-helix [12]. The specific localization of CENP-A at centromeres plays a central role in proper chromosome segregation and has been linked to cell cycle timing regulation, genome stability and cancer development [13–20]. The precise and spatiotemporal localization of CENP-A in centromeric nucleosomes is mediated by a system distinct from that used for the core histone [21–25], and the HJURP (Holliday junction recognition protein) is the dedicated deposition factor [17, 26, 27]. A domain found in the HFD of CENP-A, called the CENP-A targeting domain (CATD), mediates the specific interaction of CENP-A with HJURP and to date is the only domain identified essential for CENP-A centromeric localization [28]. Some modifications in the amino-terminus of CENP-A have recently been identified as regulatory signals for its centromeric targeting and chromosome segregation [9, 13, 29, 30]. However, the role of the amino-terminus of CENP-A is poorly understood.

Histones are synthesized in the cytoplasm, and the first step of their assembly into new chromatin is their import into the nucleus from the cytoplasm. Core histones contain a nuclear localization sequence (NLS) in the amino-terminal tail and are imported into the nucleus by members of the karyopherin (Kap)/importin family [31]. It has been determined that the positive charge of the basic acid residues in the N-tail promotes NLS function [32]. CENP-A is a unique variant that possesses a distinct protein sequence at its amino-terminus, compared with the core histones, and the molecular mechanism for the nuclear import of CENP-A has not yet been deciphered.

In this study, we examined the role of the amino-terminal tail of CENP-A. We identified two motifs to be critical for CENP-A nuclear accumulation and a third motif essential for centromeric accumulation.

## RESULTS

### A region, R^29^-K^53^, of the amino-terminus of CENP-A is required for its nuclear accumulation

**Figure 1:**
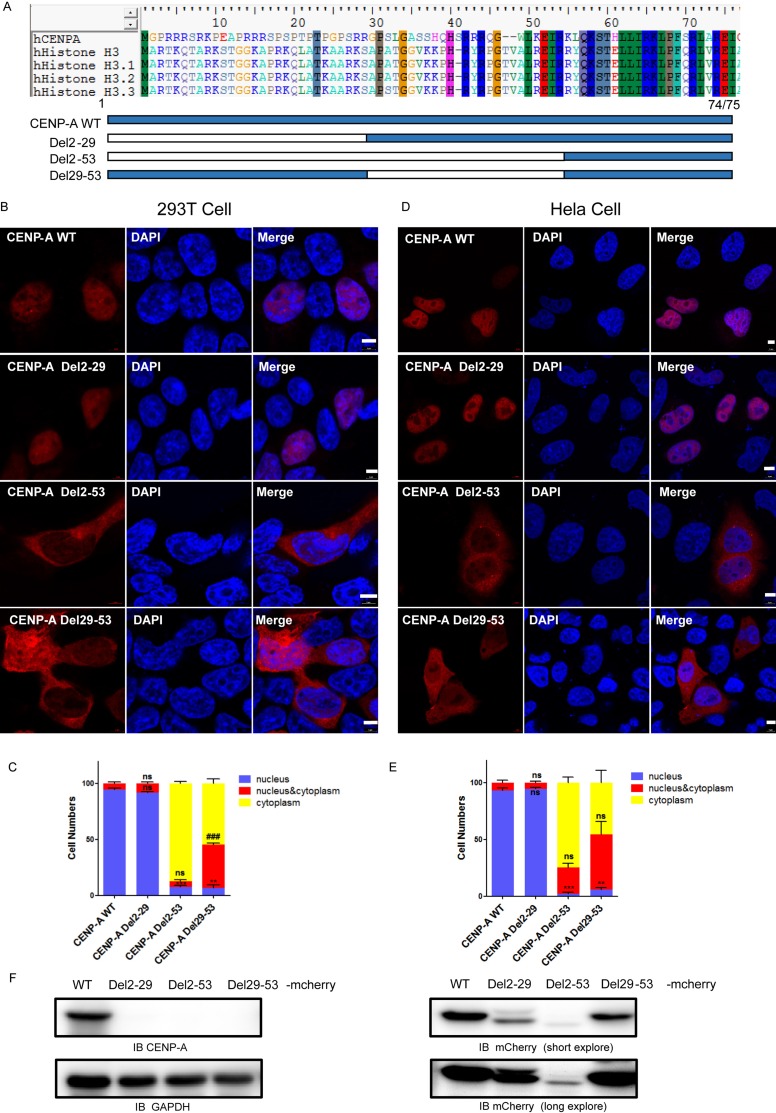
The nuclear localization of CENP-A requires its amino-terminus (**A**) Schematic drawing showing the N-tail deletion mutants of CENP-A that we generated. (**B, D**) The localization of wild-type CENP-A and the mutants in 293T (B) and HeLa (D) cells. Scale bar is 5 μm. C, D, Statistical results from 100 of the 293T (**C**) and HeLa (**E**) mCherry-positive cells. Data are presented as the mean ± SEM. Nuclear localization (compared to wild type), **p < 0.01, ***p < 0.001. nucleus/cytoplasm localization (compared to wild type), ^##^*p* < 0.01, ^###^*p* < 0.001. ns, no significance. (**F**) Western blotting confirmed the expression of mCherry-tagged CENP-A protein in 293T cells.

CENP-A possesses an HFD quite similar to that of the canonical histone H3, but it varies at the amino-terminus. To examine the role of the amino-terminus of CENP-A, we first generated two deletion mutants of CENP-A, Del2-29 and Del2-53, which contained deletions of G^2^-R^29^ and G^2^-K^53^, respectively (Figure 1A). The wild type and mutant proteins were fused to mCherry at the C-terminal end to facilitate visualization of their cellular localization in both 293T and HeLa cells [17, 26]. The expression of wild-type and mutant CENP-A-mCherry in 293T cells was confirmed by both CENP-A and mCherry antibody because the deletion mutant cannot be recognized by CENP-A antibody (Figure 1F). Compared to the exclusive nuclear localization of wild-type CENP-A (Figure 1B and 1D, first rows), the mutant Del2-29 localized to nucleus (Figure 1B and 1D, second rows), and the mutant Del2-53 mainly localized to the cytoplasm (Figure 1B and 1D, third rows). The results suggested that the region R^29^-K^53^ is required for CENP-A nuclear import. To further confirm this, a mutant with the region R^29^-K^53^ deleted, Del29-53, was generated (Figure 1A). The Del29-53 mutant localized to both the cytoplasm and the nucleus (Figure 1B and 1D, bottom rows). More than 100 mCherry-positive 293T or HeLa cells were examined for each group, and the localization results were plotted and compared (Figure 1C, 1E).

### Two motifs in the region R^29^-K^53^, specifically R^42^R^43^R^44^ and K^49^R^52^K^53^K^56^, are required for CENP-A nuclear accumulation

**Figure 2:**
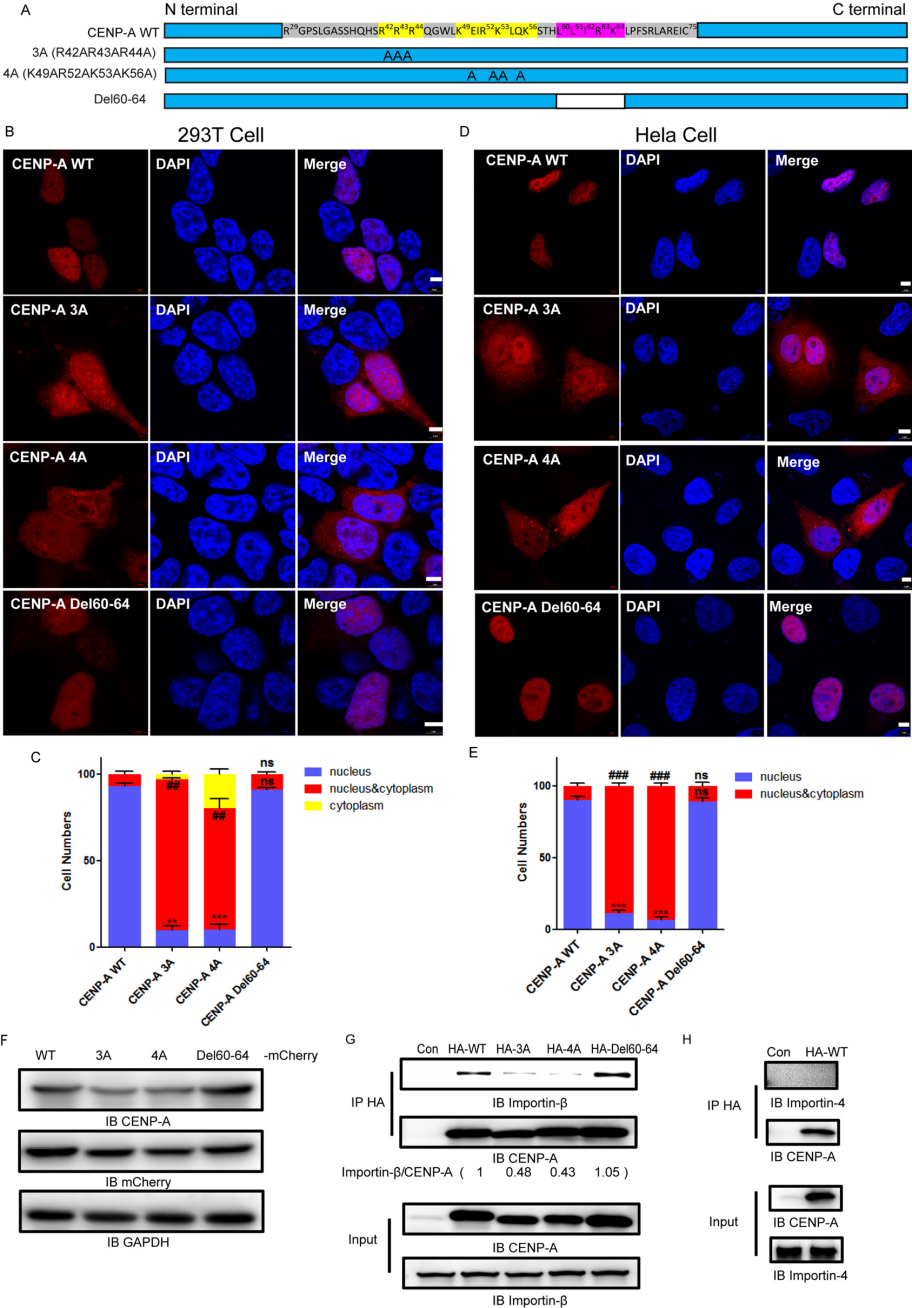
Two motifs, R^42^R^43^R^44^ and K^49^R^52^K^53^K^56^, are required for nuclear accumulation of CENP-A (**A**) A schematic drawing showing the amino acid constituents of R^29^-C^75^. Three mutants, R42A R43A R44A (3A), K49A R52A K53A K56A (4A) and Del60-64, were generated. (**B**, **D**), The localization of wild-type CENP-A and mutants 3A, 4A and Del60-64 in 293T (B) and HeLa cells (D). Scale bar is 5 μm. C, (**E**) Statistical results from 100 mCherry-positive 293T (**C**) and HeLa (D) cells. Nuclear localization (compared to wild type), **p < 0.01, ***p < 0.001. nucleus/cytoplasm localization (compared to wild type), ^##^*p* < 0.01, ^###^*p* < 0.001. ns, no significance. (**F**) Western blotting confirmed the expression of mCherry-tagged CENP-A and mutant proteins in 293T cells. (**G**) CENP-A interacts with endogenous Importin-β. (**H**) CENP-A does not interact with Importin 4. The western blotting images were quantitated using FluorChem FC3 software.

We examined the amino residues of the region R^29^-K^53^ (Figure 2A). There are two motifs in the region, R^42^R^43^R^44^ and K^49^R^52^K^53^K^56^, that contain tandem repeats of polar basic residues, typical of a protein nuclear localization signal [33]. We noticed that there was a leucine-rich motif, L^60^L^61^I^62^R^63^K^64^L^65^, which is typical of a protein nuclear export signal (NES), close to the R^29^-K^53^ region [34]. We generated three mutants, R42A R43A R44A (3A), K49A R52A K53A K56A (4A) and Del60-64, in which L^60^-K^64^ was deleted (Figure 2A). The expression of the 3A, 4A and Del60-64 mutants in 293T cells was confirmed by both CENP-A and mCherry antibodies (Figure 2F). In contrast to the exclusive nuclear localization of wild type CENP-A (Figure 2B and 2D, first rows), the mutants 3A (Figure 2B and 2D, second rows) and 4A (Figure 2B and 2D, third rows) were distributed in both the nucleus and the cytoplasm in both 293T and HeLa cells. The Del60-64 mutant (Figure 2B and 2D, bottom rows) was localized to the nucleus as efficiently as the wild-type protein. More than 100 cells for each group were examined to confirm the observation (Figure 2C and 2E). The results suggested that the motifs R^42^R^43^R^44^ and K^49^R^52^K^53^K^56^ are required for CENP-A nuclear accumulation, and the motif L^60^L^61^I^62^R^63^K^64^ is not involved in its cytoplasm/nuclear localization.

Previous reports have suggested that the core histones are imported into the nucleus by members of the importin family [35–37]. We explored whether importins might be responsible for the nuclear import of CENP-A. Protein co-immunoprecipitation assays suggested that endogenous Importin-β (Figure 2G), but not Importin-4 (Figure 2H), co-immunoprecipitated with CENP-A. More importantly, the results showed that the interactions of mutants 3A or 4A with Importin-β were significantly reduced (Figure 2G). The mutant Del60-64 interacted with Importin-β as efficiently as the wild type protein. These results suggested that Importin-β interacts with the two motifs, R^42^R^43^R^44^ and K^49^R^52^K^53^K^56^, and potentially mediates the nuclear import of CENP-A.

**Figure 3:**
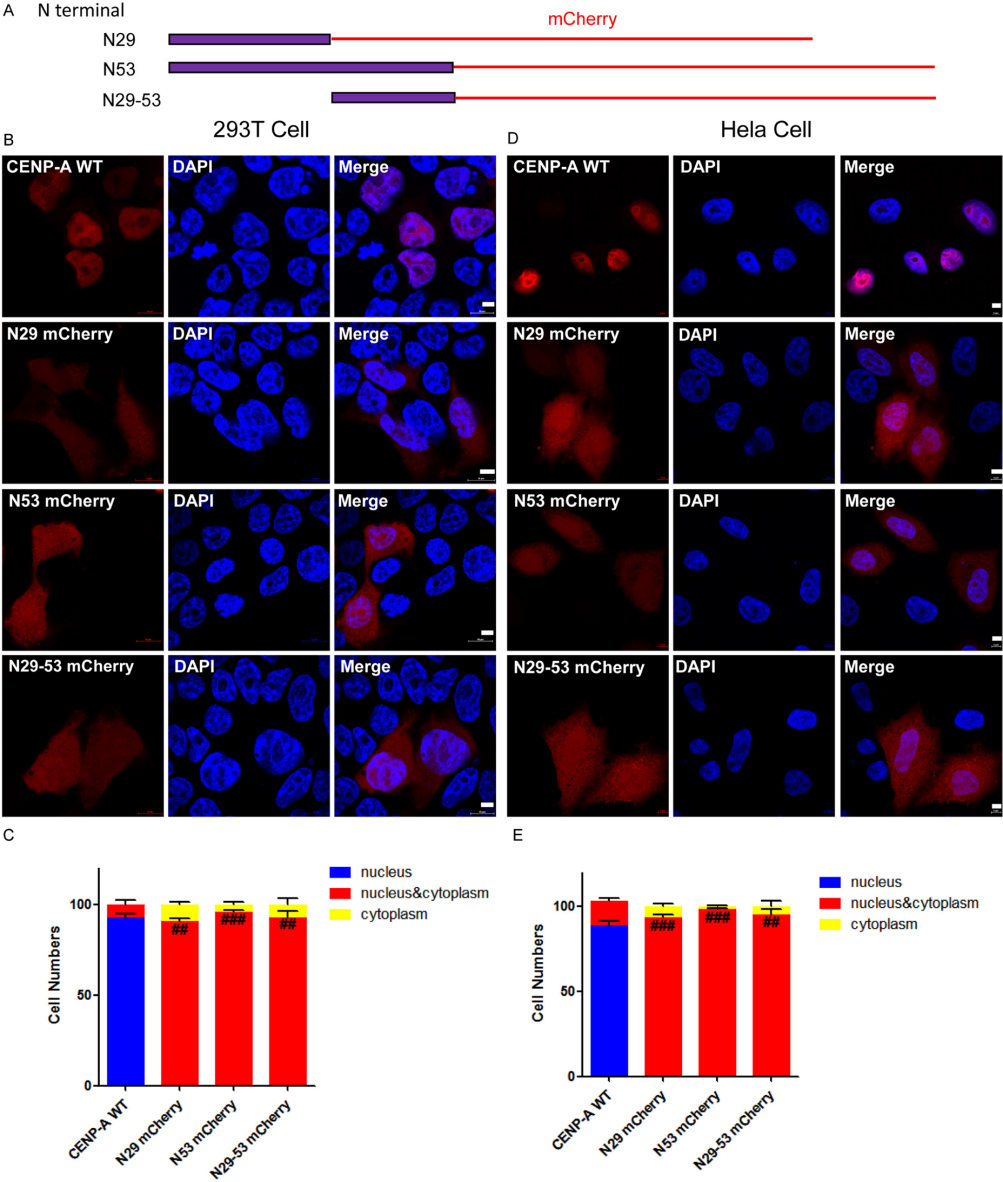
Evaluation of the ability of the amino-terminus of CENP-A to target CENP-A to the nucleus (**A**) Different regions, the first 29, the first 53 or the 29^th^-53^rd^ amino acid residues, of the amino-terminus of CENP-A were fused to mCherry (N29-mCherry, N53-mCherry or N29-53-mCherry). (**B**, **D**) Fused mCherry proteins were expressed in both 293T and HeLa cells. Scale bar is 5 μm. (**C**, **E**) Statistical results from 100 of the 293T (C) and HeLa (E) mCherry-positive cells. ^##^*p* < 0.01, ^###^*p* < 0.001 (nucleus/cytoplasm localization compared to wild-type CENP-A-mCherry).

To clarify whether the amino-tail of CENP-A is sufficient for nuclear targeting, we fused the amino-tail of CENP-A to mCherry (Figure 3A) and expressed the fusion protein in both 293T and HeLa cells (Figure 3B and 3D). More than 100 mCherry-positive 293T or HeLa cells were examined for each group, and the localization results were plotted and compared (Figure 3C and 3E). The data revealed that the amino-tail alone is not sufficient for targeting CENP-A to the nucleus and suggested that there are other elements beyond the amino-tail that are also required for CENP-A nuclear localization.

### The two motifs R^42^R^43^R^44^ and K^49^R^52^K^53^K^56^ of CENP-A are not involved in CENP-A localization to the centromere

**Figure 4:**
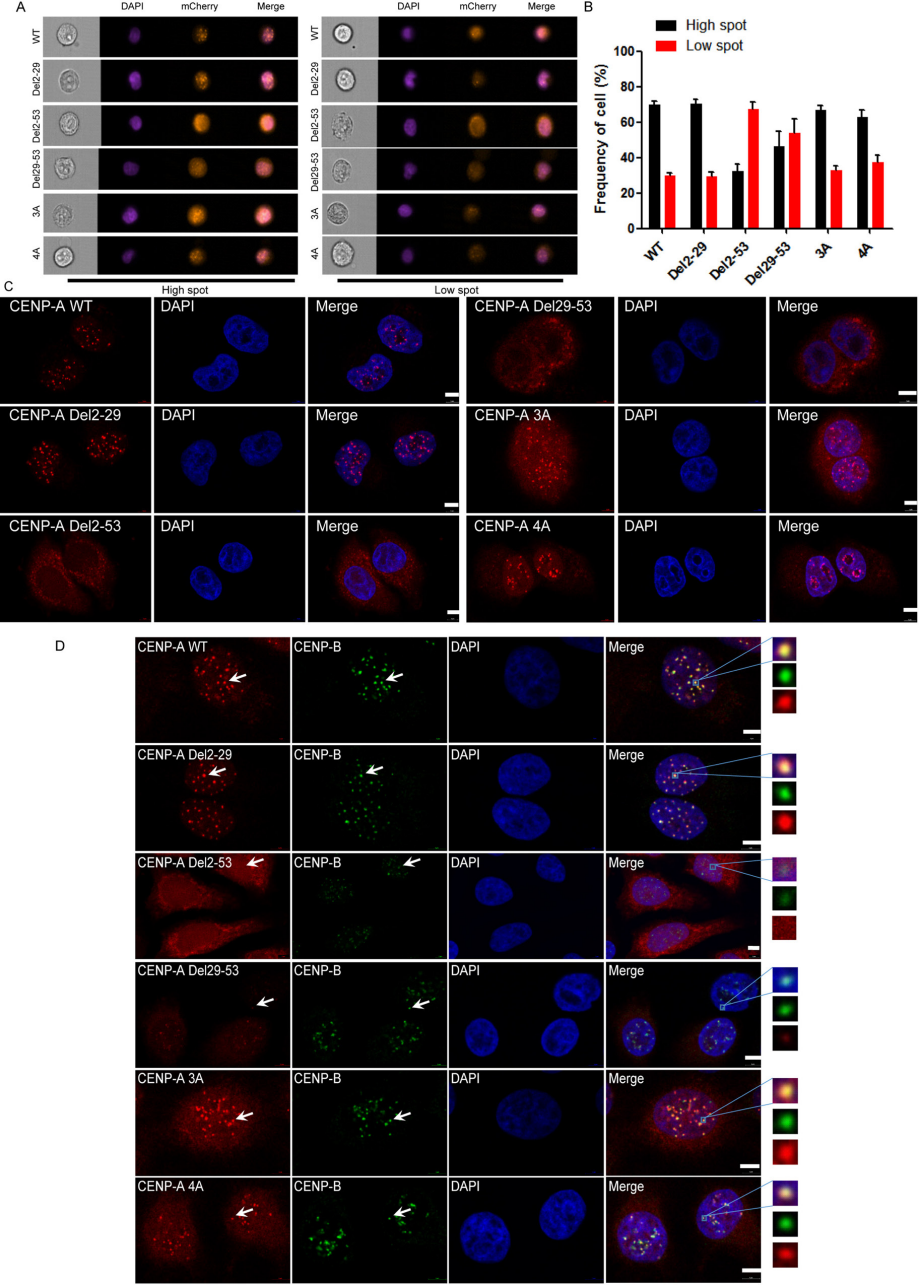
R^42^R^43^R^44^ and K^49^R^52^K^53^K^56^ were not involved in the centromeric localization of CENP-A (**A**) Representative pictures of the high-spot and low-spot population of HeLa cells expressing mCherry-fused CENP-A and its mutants acquired with Amnis^®^ ImageStream^x^ Mark II flow cytometry. (**B**) Statistical analysis of the high-spot and low-spot population of CENP-A and its mutants. (**C**) Centromeric localization of wild-type CENP-A and Del2-29, 3A and 4A mutant proteins. Scale bar is 5 μm. (**D**) Localization of the endogenous centromeric marker protein CENP-B and wild-type CENP-A, Del2-29, 3A and 4A mutant proteins. Scale bar is 5 μm.

We noticed that mutants Del29-53, 3A and 4A were not exclusively cytoplasmic, and there was a significant nuclear distribution of these mutated proteins. Given that CENP-A is a structural and functional component of the centromere, we asked whether these mutants are also defective in centromere targeting. To do this, we generated lentiviruses for mCherry-fused wild-type CENP-A and its mutants and infected HeLa cells to obtain stable expression of these proteins. We examined whether these mutated proteins were targeted to the centromere with both the ImageStream system and microscopy. In the imaging flow cytometer, cells in each group were classified into one of two categories, either high-spot or low-spot. In the high-spot population, the mCherry signal presented as many discrete dots, which is the typical localization pattern of a centromeric protein (Figure 4A, left panel), and in the low-spot group, less discrete dots were presented (Figure 4A, right panel). The results obtained from imaging flow cytometer suggested that G^2^-R^29^ and the two motifs R^42^R^43^R^44^ and K^49^R^52^K^53^K^56^ are not involved in centromeric accumulation because they behaved similarly to wild type (Figure 4B). The localization to the centromere of Del2-53 and Del29-53 was greatly impaired, primarily because they are defective in nuclear import. The cellular localization of each mutant under microscopy (Figure 4C) was consistent with that obtained from the ImageStream system. The data suggested that G^2^-R^29^, R^42^R^43^R^44^ and K^49^R^52^K^53^K^56^ of CENP-A are not involved in CENP-A localization to the centromere.

To further confirm whether the dots were localized to the centromere, we visualized an endogenous centromeric marker, CENP-B, using immunofluorescence (Figure 4D). The dots in the mutants Del2-29, 3A and 4A co-localized very well with CENP-B, as did wild-type CENP-A (Figure 4D). There was no signal overlap with CENP-B for the mutants Del2-53 and very weak signal for the mutant Del29-53 (Figure 4D). The data suggested that the mutants Del2-29, 3A and 4A are functionally intact in regard to targeting to the centromere, and the motifs involved in CENP-A nuclear accumulation are not involved in its centromeric accumulation.

### A new motif, L^60^-I^62^, is involved in CENP-A centromeric accumulation and H4 association

**Figure 5:**
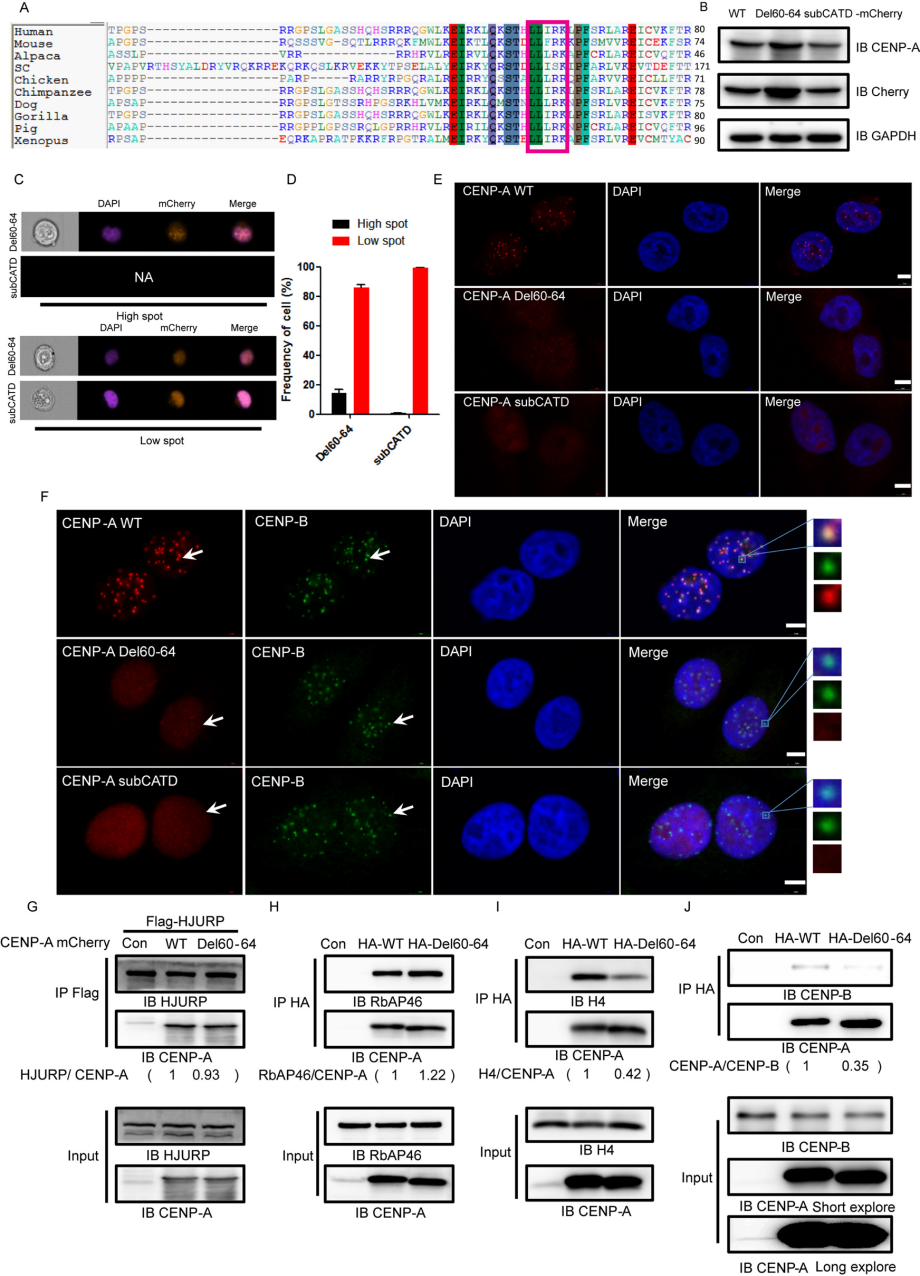
A new motif, L^60^L^61^I^62^R^63^K^64^, is required for CENP-A centromeric localization (**A**) The protein sequence alignment of the centromere-specific histone variants of histone H3 from different species showing conservation of the L^60^L^61^I^62^R^63^K^64^ motif. (**B**) The expression of mCherry-tagged wild-type CENP-A and the mutant proteins Del60-64 and subCATD in HeLa cells. (**C**) Representative pictures of the high-spot and low-spot populations of HeLa cells expressing mCherry-fused CENP-A and mutant proteins acquired with ImageStream cytometry. (**D**) The statistical analysis results of high-spot and low-spot populations of CENP-A and mutants. (**E**) Del60-64 partially lost centromeric localization,. (**F**) Localization of the endogenous centromeric marker protein CENP-B and mCherry-tagged CENP-A, Del60-64, and subCATD. Scale bar is 5 μm. (**G**–**J**) The association of CENP-A or Del60-64 with HJURP (G), RbAp46 (H), Histone H4 (I) and CENP-B (J).

We found that the mutant Del60-64, in which L^60^L^61^I^62^R^63^K^64^ was deleted, localized to the nucleus as efficiently (Figure 2B and 2D, bottom panels) as wild-type CENP-A. A protein sequence alignment of the centromere-specific histone variants of histone H3 from different species suggested that this region is relatively conserved (Figure 5A). The mutant Del60-64 was expressed in HeLa cells to a similar level as wild-type CENP-A and a CATD mutant, subCATD, in which the CATD of CENP-A is substituted with the corresponding region of the core Histone H3 (Figure 5B). The CATD mutant, subCATD, is known to be defective in centromeric localization (Figure 5C–5E) [38]. We did detect very weak centromeric localization with the mutant Del60-64 (Figure 5C–5E). The centromeric localization of the mutant Del60-64 was greatly impaired compared to wild type. The CENP-B co-localization assay yielded the same results (Figure 5F). The data suggested that the motif L^60^L^61^I^62^R^63^K^64^ is required for CENP-A centromeric accumulation.

To investigate why the mutant Del60-64 lost its ability to localize to the centromere, we examined its interaction with HJURP and RbAp46, both of which are involved in targeting CENP-A to the centromere. We found that the interaction of Del60-64 with HJURP (Figure 5G) and RbAp46 (Figure 5H) was not affected. Interestingly, we found that its interaction with Histone H4 or with CENP-B was significantly reduced (Figure 5I and 5J). The results suggested that the motif L^60^L^61^I^62^R^63^K^64^ is potentially involved in the association of CENP-A with the core Histone H4.

**Figure 6:**
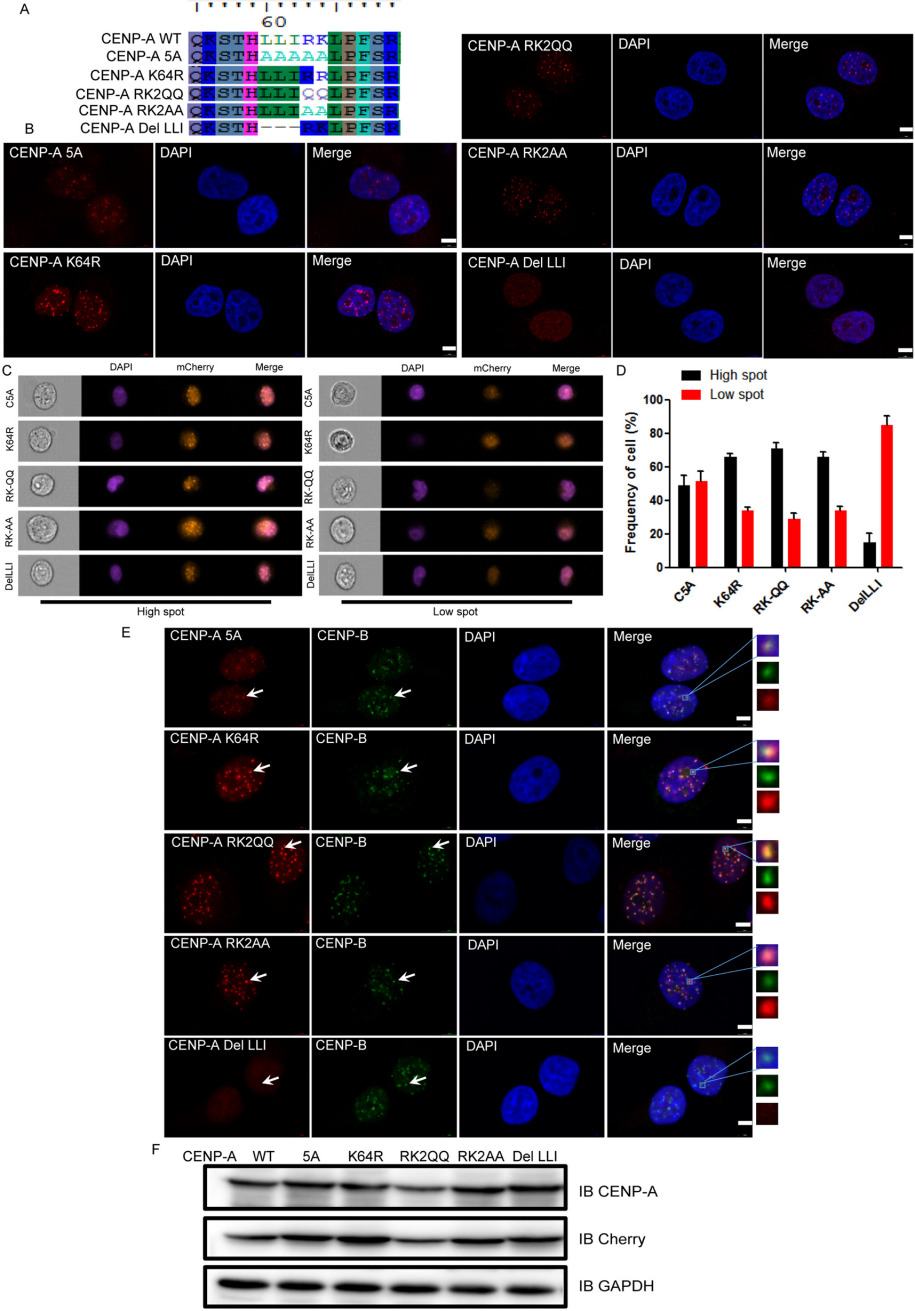
L^60^L^61^I^62^ are the most important residues for CENP-A centromeric localization within the L^60^L^61^I^62^R^63^K^64^ region (**A**) A protein sequence alignment showing the mutants we generated within the motif L^60^L^61^I^62^R^63^K^64^. (**B**) The centromeric localization of the mutant DelLLI was greatly impaired when compared to other mutants with mutation within the region L^60^-K^64^. Scale bar is 5 μm. (**C**) Representative pictures of high-spot and low-spot populations of HeLa cells expressing mCherry-fused CENP-A and its mutants acquired with ImageStream cytometry. (**D**) The statistical analysis results of high-spot and low-spot populations of CENP-A and its mutants. (**E**) Localization of CENP-B and CENP-A mutants. Scale bar is 5 μm. (**F**) The expression of mCherry-tagged CENP-A proteins in HeLa cells.

There are two types of amino acid residues within the L^60^L^61^I^62^R^63^K^64^ sequence, the nonpolar and hydrophobic residues Leu/Ile and the polar basic residues Arg/Lys. We evaluated the role of these Leu/Ile and Arg/Lys residues in the centromeric localization of CENP-A. In total, 5 mutants were generated: L60A L61A I62A R63A K64A (5A), K64R, RK2QQ, RK2AA, and DelLLI (in which L^60^L^61^I^62^ were deleted) (Figure 6A). Each of these mutants expressed intact protein that was recognized by CENP-A and mCherry antibodies (Figure 6F). Mutating five of the residues to Ala impaired but did not abrogate the CENP-A centromeric accumulation. The polar basic residues R^63^ and K^64^ played a minor role in CENP-A centromeric accumulation because the mutation of these two residues had no effect on the centromeric localization of CENP-A (Figure 6B–6D). However, deletion of L^60^L^61^I^62^ (DelLLI in Figure 6B–6D) abrogated the centromeric accumulation of CENP-A. The CENP-B co-localization assay yielded the same results (Figure 6E). These results underscored the critical role of the nonpolar and hydrophobic residues L^60^L^61^I^62^ in this motif in CENP-A centromeric accumulation.

### The effect of CENP-A mutants on cell cycle and mitotic arrest

**Figure 7:**
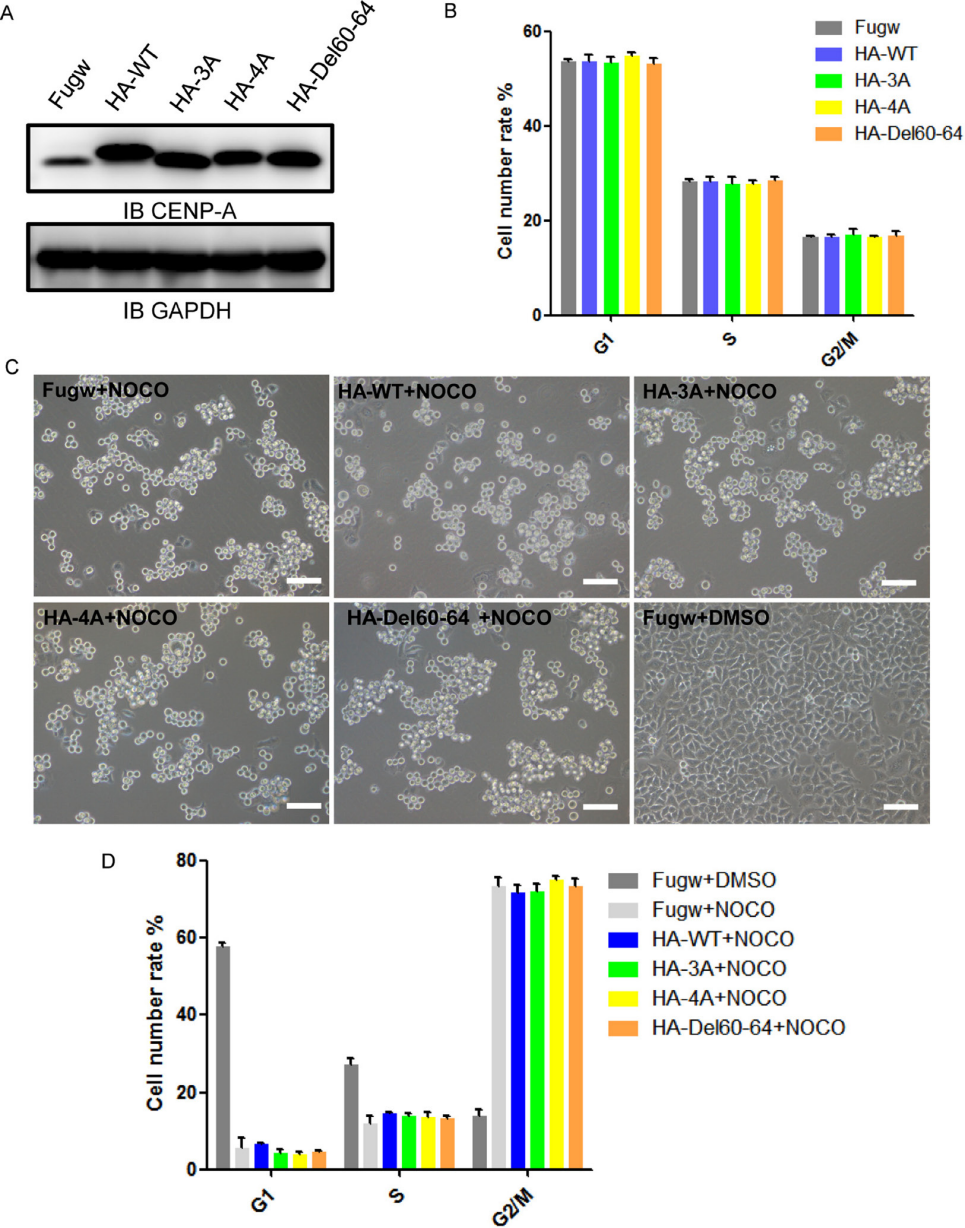
The effect of CENP-A mutants on cell cycle and mitotic arrest (**A**) Western blotting confirmed the expression of HA-tagged wild-type CENP-A and mutant proteins in HeLa cells. (**B**) Cell cycle analysis of HeLa cells with the expression of wild-type CENP-A and mutant proteins for 72 hours. (**C**) Cells in mitotic arrest expressing wild-type CENP-A and mutants in HeLa cells. Cells were observed using microscopy after treatment with 100 ng/ml nocodazole for 18 hours. Scale bar is 100 μm. (**D**) The percentages of the G1, S and G2/M populations after 100 ng/ml nocodazole treatment for 18 hours were determined using PI staining and FACS analysis.

We were interested in whether mutant CENP-A has any physiological impact on cellular function. We expressed the mutant in HeLa cells and examined cell cycle progression and mitotic arrest upon the mitotic insult. Western blotting confirmed that the expression levels of HA-tagged CENP-A wild-type and mutant proteins in HeLa cells were similar (Figure 7A). The cell cycle progression was not altered upon the expression of these CENP-A mutants (Figure 7B). The cells with the expression of mutant and wild-type CENP-A responded similarly to the mitotic insult induced by the microtubule depolymerizer nocodazole (Figure 7C and 7D). Overall, the effects of the mutant CENP-A on cell cycle progression and mitotic arrest were minor in the current experimental setting. Expression of the mutant in the context of endogenous CENP-A depletion could be helpful in evaluating the effect of these mutants.

## DISCUSSION

Histones and histone variants are synthesized in the cytoplasm, and nuclear import of histones is a prerequisite for the downstream deposition to form chromatin, which is important for the efficient progression of the cell cycle [39]. In yeast, it has been demonstrated that each core histone contains an NLS located at their amino terminus [35, 36]. The minimal NLS domains of H3 and H4 have been mapped to the initial residues, residues 1–28 for Histone H3 and residues 1–21 for Histone H4 [36]. The NLS of CENP-A has never been reported. We identified two motifs in CENP-A, R^42^R^43^R^44^ and K^49^R^52^K^53^K^56^, both of which consist of basic amino acid residues, to be critical for CENP-A accumulation in the nucleus. Distinct from the NLSs of Histone H3/H4, which are located at the very beginning of their amino-termini, the two motifs essential for CENP-A nuclear accumulation are located in the region R^29^-K^53^. The initial residues 1–28 of CENP-A are not involved in its nuclear import. To our surprise, the two motifs are required but not sufficient for CENP-A nuclear accumulation (data not shown). There are other elements beyond these two motifs that participate in the nuclear import of CENP-A. In the current structural model of the CENP-A nucleosome, these two motifs are located preceding to or in the αN helix of CENP-A, which functions in contacting DNA and stabilizing the conventional nucleosomal DNA ends in the nucleus [9, 10]. Our findings suggest that in the cytoplasm, this region of CENP-A mediates the interaction with importin for nuclear targeting [40]. This highlights the dual functions of the CENP-A amino terminus, participating in nuclear targeting in the cytoplasm and stabilizing DNA binding in the centromere nucleosome.

The motif L^60^L^61^I^62^ is located in the region between the αN-helix and the α1-helix, and its function has not been defined yet [10, 11, 41]. We found that this motif is critical for centromeric accumulation of CENP-A. Deletion of this motif greatly impaired the centromeric localization of CENP-A. The CATD of CENP-A is the only unique region identified to date that is required for CENP-A localization. The motif L^60^L^61^I^62^ is a newly discovered site allowing efficient incorporation of CENP-A into centromeric chromatin. The CATD mediates the specific interaction of CENP-A and HJURP [17, 26, 49] for centromeric targeting. However, we found that this motif does not mediate the interaction of CENP-A with its centromeric targeting molecules HJURP and RbAp46 [17, 26]. The results suggest that this motif is involved in CENP-A association with the core histone H4, which is mediated by the α2-L2-α3 segment of CENP-A. The L^60^L^61^I^62^ motif is a novel region at the amino-terminus of CENP-A that is potentially required for assembly of the CENP-A-H4 heterodimer. This observation is consistent with the report that the deposition of CENP-A requires formation of the CENP-A-H4 heterodimer to provide a specific recognition site for HJURP binding [11, 41].

Our study characterized the key region and residues at the amino-terminus of CENP-A that are critical for nuclear accumulation, CENP-A/H4 assembly and centromere accumulation. These findings underscore the multiple functions and importance of the flexible amino-terminus of CENP-A [9]. Our study facilitates improved understanding of the behavior of CENP-A in cells.

## MATERIALS AND METHODS

### Cell culture and chemical treatment

The 293T and HeLa cell lines were cultured in Dulbecco's modified Eagle's medium (DMEM; GIBCO, Grand Island, USA) supplemented with 10% fetal bovine serum (ExCell Bio, Shanghai, China), 100 U/ml streptomycin and 100 U/ml penicillin. Cells were cultured at 37°C in a humidified incubator under 5% CO_2_. For drug treatment, cells were treat with Paclitaxel or nocodazole (S1150, S2775; Selleckchem, Houston, USA) for 18 hours.

### Plasmids, transfections and virus generation

The human CENP-A (Gene ID: 1058) coding sequence was amplified using PCR (E003-01A, Novoprotein) and substituted for the H2B coding sequencing in the PGK-H2BmCherry plasmid (Addgene Plasmid #21217) with the recombinase NR001A (Novoprotein). This generated an mCherry-fused CENP-A overexpression plasmid. CENP-A mutants were generated using a QuikChange kit from Agilent and verified by DNA sequencing. Transient transfections of the DNA were performed using a transfection reagent from Exelgene (USA) as described in [43]. Virus generation and the infection procedure were conducted as previously described [44–46].

### Western blotting, immunofluorescence staining, immunoprecipitation and antibodies

Western blotting, immunoprecipitation and immunofluorescence were performed as previously described [44–47]. For immunoprecipitation, cell lysates were obtained by sonication in lysis buffer (50 mM Tris (pH 7.4), 150 mM NaCl, 1 mM EDTA, 1% (v/v) NP-40 alternative, and 15% (v/v) glycerol). Prior to use, the buffer was supplemented with complete protease inhibitor cocktail (Roche) and 2 mM sodium fluoride and 1 mM sodium orthovanadate (Na3 VO4, Sigma) as phosphatase inhibitors. The cell lysates were incubated with antibody and Protein A/G beads (Sigma) for 4 hours. The beads were washed with lysis buffer three times and the immunoprecipitates were analyzed by western blotting. For immunofluorescence staining, cells plated on a glass coverslip were fixed with 4% paraformaldehyde for 15 min, rinsed in PBS, treated with 0.2% Triton X-100 for 10 min, washed with PBS, blocked with 10% FBS for 1 hour, then incubated with primary antibody, and washed with PBS. Then, the cells were incubated with dye-conjugated secondary antibody and were finally mounted using mounting medium with DAPI. All the western blotting, immunoprecipitations and immunofluorescence staining were repeated three times independently, and the following antibodies were used: anti-CENP-A (Cell Signaling Technology, CST2186), anti-CENP-B, (ab25734), anti-Importin4 (ab181037), anti-Importin-β/NTF-97 (ab45938), anti-RbAP46 (Abcam, ab109285), anti-HJURP (Santa Cruz, sc-168091), anti-RbAp46 (ab28500), anti-GAPDH (ABclonal), anti-HA (Bioworld; Nanjing, China; AP0005M), anti-Flag (Sigma, F1804), and horseradish peroxidase (HRP)-conjugated secondary antibodies (Cell Signaling Technology). In addition, Alexa Fluor-conjugated secondary antibodies were obtained from Invitrogen (Eugene, OR. USA). A Leica TSC SP5 microscope was used for imaging (Leica Microsystems, Bensheim, Germany). The western blotting images were quantitated with FluorChem FC3 software. Analysis was performed using a Student's *t-test* or One-way analysis of variance (ANOVA). Values of *P* < 0.05 were considered to be statistically significant.

### Imagestream

Amnis^®^ ImageStream^x^ Mark II is a multispectral flow cytometer that combines standard microscopy with flow cytometry. It acquires up to 100 cells/sec, with simultaneous acquisition of six images of each cell including bright field, scatter, and multiple fluorescence images. Cells were fixed with 70% cold-ethanol, stained with propidium iodide and subjected to Amnis^®^ ImageStream^x^ Mark II flow cytometry for analysis of nuclear/cytoplasm and centromeric localization [48, 49].
